# Patient satisfaction with nursing care in Ethiopia: a systematic review and meta-analysis

**DOI:** 10.1186/s12912-019-0348-9

**Published:** 2019-07-08

**Authors:** Henok Mulugeta, Fasil Wagnew, Getenet Dessie, Henok Biresaw, Tesfa Dejenie Habtewold

**Affiliations:** 1 Lecturer of Nursing, Department of Nursing, College of Health Science, Debre Markos University, P.O. Box 269, Debre Markos, Ethiopia; 2 Lecturer of Nursing, Department of Nursing, School of Health Science, College of Medicine and Health Science, Bahir Dar University, P.O. Box 79, Bahir Dar, Ethiopia; 3 Lecturer of Nursing, Department of Nursing, College of Health Science, University of Gondar, P.O. Box 196, Gondar, Ethiopia; 4 Department of Epidemiology, University of Groningen, University Medical Center Groningen, Groningen, the Netherlands

**Keywords:** Nursing care, Patient satisfaction, Systematic review, Meta-analysis, Ethiopia

## Abstract

**Background:**

Patient satisfaction with nursing care has been considered as the most important predictor of the overall patient satisfaction with hospital service and quality of health care service at large. However, the national level of patient satisfaction with nursing care remains unknown in Ethiopia. Hence, the objective of this systematic review and meta-analysis was to estimate the level of patient satisfaction with nursing care and its associated factors in Ethiopia.

**Methods:**

Studies were accessed through an electronic web-based search strategy from PubMed, Cochrane Library, Google Scholar, Embase, PsycINFO, and CINAHL by using a combination of search terms. The quality of each included article was assessed using a modified version of the Newcastle-Ottawa Scale for cross-sectional studies. All statistical analyses were done using STATA version 14 software for windows, and meta-analysis was carried out using a random-effects method. The Preferred Reporting Items for Systematic Reviews and Meta-Analyses (PRISMA) guideline was followed for reporting results.

**Results:**

Of 1166 records screened, 15 studies with 6091 patients fulfilled the inclusion criteria and were included in the meta-analysis. The estimated pooled level of patient satisfaction with nursing care in Ethiopia was 55.15% (95% CI (47.35, 62.95)). Patients who have one nurse in charge (OR: 1.08, 95% CI: 0.45–2.62, I^2^: 77.7%), with no history of previous hospitalization (OR: 1.37, 95% CI: 0.82–2.31, I^2^: 91.3%), living in the urban area (OR: 1.07, 95% CI: 0.70–1.65, I^2^: 62.2%), and those who have no comorbid disease (OR: 1.08, 95% CI: 0.48–2.39, I^2^: 91.9%) were more likely to be satisfied with nursing care compared with their counterparts although it was not statistically significant.

**Conclusion:**

About one in two patients were not satisfied with the nursing care provided in Ethiopia and may be attributed to several factors. Therefore, the Ministry of Health should give more emphasis to the quality of nursing care in order to increase patient satisfaction and improve the overall quality of healthcare service in Ethiopia.

## Background

Quality healthcare delivery and creation of patient satisfaction are the primary hospital’s goals [1]. Patient satisfaction has been described as the value and reaction of patients towards the care they received [2]. According to the American Nursing Association (ANA), patient satisfaction with nursing care is defined as patients’ value and attitude towards the care they received from the nursing staffs during their hospitalization [3].

Patient satisfaction with nursing care is considered as a fundamental indicator of quality health care service provided in hospitals and one of the ways of evaluating the performance of health care service [4, 5]. It is a multidimensional concept that has the following aspects: the art of care, the technical quality of care convenience, cost, a physical and environmental organization, availability of the resource, continuity of care and outcomes [6, 7].

Measuring the level of patient satisfaction is challenging. Patient satisfaction assessment surveys should accurately measure the patient’s reaction to the care they received using a valid and reliable instrument. Measuring patient satisfaction with the different instruments may provide different results of outcome (level of patient satisfaction) [8–10].

Nurses are a pivotal part of the health care system who spend more time with patients and provide about 80% of primary health care service in the hospital. Hence, measuring the level of patient satisfaction with nursing care is important to determine the overall satisfaction of the hospital service provided [7, 11, 12], and to evaluate whether patients’ needs and expectations are fulfilled which can help nurses to plan appropriate nursing interventions for the patients [13].

Currently, patient satisfaction is a major concern of healthcare system, particularly in developing countries [14]. Satisfied patients are more likely to have a good relationship with nurses, which suggest improved quality of care [15, 16]. Literature also suggested that patient satisfaction is directly linked to better patient outcomes. Furthermore, achieving the optimal level of patient satisfaction with nursing care results in better patient compliance with health care regimens [7].

Determining the factors that influence patient satisfaction is important for nurses to continuously improve the quality of nursing care. Patient satisfaction with nursing care can be affected by numerous factors [7, 17] including patient-related factors(e.g. residence, history of the previous hospitalization) and context-related factors (e.g. availability of assigned nurse/s, behaviors of nurses, and the surrounding physical environment) [2, 7, 13, 18, 19].

In recent years, many studies have been conducted to determine the level of patient satisfaction with nursing care. For instance, studies done in Iraq [20], Brazil [21] and Egypt [22] showed that patient satisfaction with nursing care was high. Additionally, the overall level of patient satisfaction with nursing care was 69% in Iran [23], 67% in Kenya [24], and 33% in Ghana [25]. On the contrary, the results of the study done in India revealed that most of the hospitalized patients had poor perception regarding nursing care [26].

The Ethiopian Federal Ministry of Health (FMoH) is striving to develop different national quality management guidelines and health sector development plans to increase patients’ satisfaction and improve the overall quality of the healthcare service in the country [27, 28]. Nurse professionals in Ethiopia are considered the backbone of the healthcare system, involving in-patient and outpatient care as well as hospital administration activities. Moreover, they can provide health education and home-based care services, with significant contribution to the prevention and treatment of diseases. Therefore, nurses have a unique role in determining the overall quality of healthcare services of a country [29, 30]. However, the overall quality of nursing care in Ethiopia is poor [31].

Although few studies have been conducted to assess the level of patient satisfaction with nursing care in Ethiopia, they were conducted in a specific institution with small sample size and their reports were inconsistent and inconclusive. Consequently, the national level of patient satisfaction with nursing care remains unknown. Therefore, the objective of this systematic review and meta-analysis was to estimate the national level of patient satisfaction with nursing care and investigate the influence of availability of assigned nurse in charge of individual care, residence, history of hospitalization, and the presence of comorbid diseases on patient satisfaction in Ethiopia. The findings of this study will be important to monitor and improve the quality of nursing care and to inform policymakers for areas of improvement in the health care system of the country.

## Methods

### Design and search strategy

The procedure for this systematic review and meta-analysis was designed in accordance with the Preferred Reporting Items for Systematic Reviews and Meta-Analyses (PRISMA) guidelines [32]. We searched PubMed, Cochrane Library, Google Scholar, CINAHL, Embase, and PsycINFO database for studies reporting the level of patient satisfaction with nursing care from study conception to May 2018. EndNote (version X8) reference management software for Windows was used to download, organize, review and cite the articles. We also manually searched cross-references in order to identify additional relevant articles. A comprehensive search was performed using the following search terms: “Patient satisfaction”, “satisfaction”, “determinants of patient satisfaction”, “nursing care”, and “Ethiopia”. Boolean operators like “AND” and “OR” were used to combine search terms.

### Eligibility criteria

We included studies reporting the level of patient satisfaction with nursing care among admitted patients irrespective of the type of satisfaction measurement instrument, the dimension of satisfaction assessed, and scoring system used to generate the overall score of satisfaction, and patient’s demographic characteristics. In addition, studies were included if they reported the association between patient satisfaction with nursing care and availability of assigned nurse in charge of individual care, residence, history of hospitalization, and the presence of comorbid diseases. Both published and gray literature reported in English language regardless of the date of study/publication were also included to obtain additional relevant studies and to increase the statistical power of estimated effect size. Nevertheless, articles without full-text and with poor methodological quality were excluded. Two authors (H.M. and G.D.) independently evaluated the eligibility of all retrieved studies, and any disagreement and inconsistencies during the selection of articles and data extraction were resolved by discussion and consensus.

### Outcome of the study and operational definition

The outcome of this study was the level of patient satisfaction with nursing care. Patient satisfaction with nursing care was defined as the patients’ opinion about the care they received from nurses during their hospitalization [7]. The independent variables were patient residence (rural versus urban), presence of one nurse in charge for individual care (yes versus no), history of previous admission to health facility (at least one history of hospital admission versus no previous hospitalization), and presence of comorbid diseases (presence of comorbid diseases other than the reason for admission versus no comorbid diseases).

### Data extraction and quality assessment

Data were extracted using a pre-piloted data extraction format prepared in a Microsoft Excel spreadsheet. The tool consisted of information regarding: author/s name, year of publication, study area and region, health institution, study design, type of satisfaction measurement instrument, sample size, prevalence of patient satisfaction towards nursing care, and information regarding the determinant factors. The data were extracted by three independent authors (H.M, FW, and G.D).

The quality of included studies was assessed using the Joanna Briggs Institute (JBI’s) critical appraisal checklist for prevalence studies [33]. Additionally, the methodological quality of studies was assessed using a modified version of the Newcastle-Ottawa Scale (NOS) for cross-sectional studies adapted from Modesti et al. [34]. Representativeness of the sample, response rate, measurement tool used, comparability of the subject, appropriateness of the statistical test used to analyze the data are some of the key criteria in Newcastle –Ottawa scale. Two authors (HM and HB) independently assessed the quality of each article. Whenever it was necessary a third reviewer (TDH) was involved. Any disagreement was resolved through discussion and consensus.

### Statistical analysis

The extracted data were imported to STATA version 14 for meta-analysis. A meta-analysis of the level of patient satisfaction with nursing care was carried out using a random-effects (DerSimonian and Laird) method since it is the most common method in a meta-analysis to adjust for the observed variability [35, 36]. The influence of selected determinant factors was also independently analyzed. The pooled effect size (i.e. proportion and odds ratio (OR)) with a 95% confidence interval (CI) was generated and presented using a forest plot. Heterogeneity across studies was evaluated using *I*^2^ statistics and Cochran’s Q test. *I*^2^ statistics is used to quantify the percentage of the total variation in study estimate due to heterogeneity. I^2^ value ranges between 0 and 100% whereby I^2^ > =75% indicate high heterogeneity across the studies. A *p*-value of less than0.05 was used to declare a statistically significant heterogeneity [37, 38]. Furthermore, the source of heterogeneity was assessed using meta-regression.

A funnel plot was used for visual assessment of publication bias. Asymmetry of the funnel plot is an indicator of potential publication bias [39]. Egger’s test was used to determine if there was significant publication bias, and a *p*-value less than 0.05 was considered to indicate the presence of significant publication bias [40]. Finally, sensitivity analysis was performed to evaluate whether the pooled effect size was influenced by individual studies. All data manipulation and statistical analyses were performed using Stata version 14.0 software for Windows.

## Results

### Search result and study characteristics

**Fig. 1:**
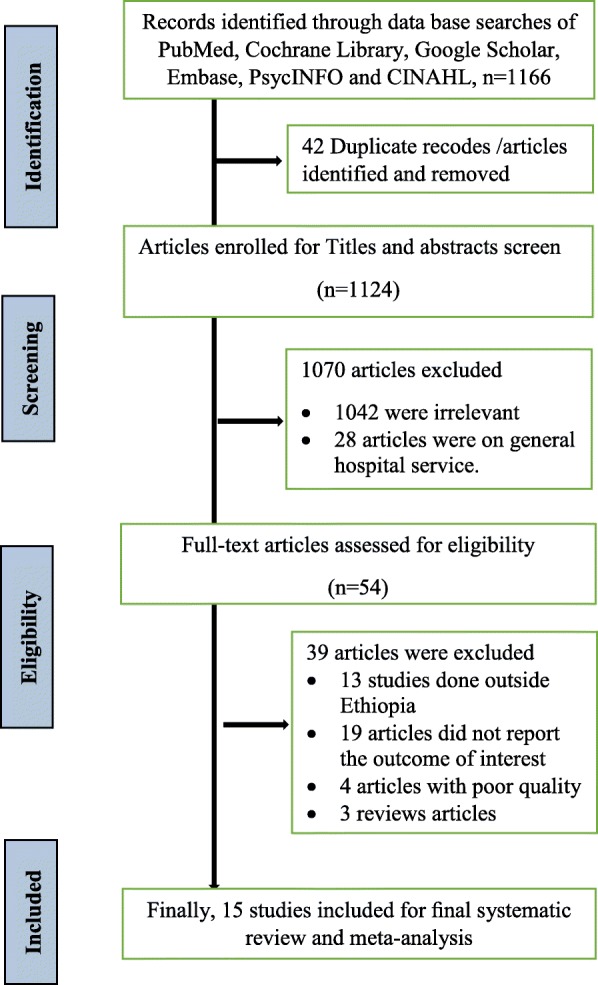
PRISMA Flowchart diagram of the study selection

The electronic online search yielded 1166 records, of which 42 duplicate records were identified and removed. Title and abstract screening resulted in the exclusion of 1042 irrelevant articles. From the remaining 82 articles, 28 articles were excluded since they reported patient satisfaction with the general hospital services. Then, 54 articles underwent for full-text review. Among these, 39 articles were excluded based on the predetermined eligibility criteria. Finally, a total of 15 articles were included in the meta-analysis (Fig. 1).

A total of 15 studies with 6091 participants were included in this meta-analysis. Of those, five studies [19, 41–44] were conducted in Addis Ababa, five [27, 45–48] in Amhara region, two [28, 49] in SNNP region, and three [18, 50, 51] in other regions (Oromia, Harari and Tigray). All the included studies were cross-sectional by design and were conducted among admitted adult patients in different hospitals of Ethiopia. Regarding instruments, twelve studies [18, 19, 27, 28, 41–48] used Newcastle satisfaction with nursing care scale (NSNS), two [49, 50] used inpatient patient satisfaction questionnaire (IPSQ) and one study [51] used patient perception of nursing care scale (PPSNS) to measure the level of patient satisfaction with nursing care (Table 1). NSNS is a standard scale with 19 items to measure the multidimensional aspect of nursing care, such as attention, availability, openness, reassurance, individual treatment, information, professionalism, knowledge, ward, and environmental management [55–57]. Participants rated their satisfaction with any aspect of nursing care, using a five-point Likert scale range (1: not at all satisfied, 2: barely satisfied, 3: quite satisfied, 4: very satisfied, 5: completely satisfied). IPSQ is adapted from NSNS that measures the perceived patient satisfaction with nursing care using a five-level Likert scale range (1: not at all satisfied, 2: barely satisfied, 3: quite satisfied, 4: very satisfied, 5: completely satisfied) [49, 50]. PPSNS assesses patient satisfaction with nursing care in terms of nursing characteristics, care related issues, information given, and caring environment [51].

**Table 1:** Characteristics of studies included in the meta-analysis of patient satisfaction with nursing care

S. No	Author/s [reference]	Publication year	Study area, region	Health facility name	Study design	Instrument	Sample size	Proportion of satisfied patients % (95%CI)
1	Mulugeta M. et al. [44]	2014	Addis Ababa	Black Lion Hospital	Cross-sectional	NSNS	374	90.1 (87.1,93.1)
2	Getachew G. et al. [43]	2016	Addis Ababa	Menelik Hospital	Cross-sectional	NSNS	372	46.7 (41.6,51.8)
3	Solomon B. [41]	2009	Addis Ababa	Addis Ababa Public Hospitals	Cross-sectional	NSNS	435	56.3 (51.6,61.0)
4	Bekele C. [42]	2005	Addis Ababa	Addis Ababa Public Hospitals	Cross-sectional	NSNS	631	67.0 (63.3,70.7)
5	Melsew G. [19]	2017	Addis Ababa	Addis Ababa Public Hospitals	Cross-sectional	NSNS	422	48.8 (44.0,53.6)
6	Melesse B. [52]	2016	Bahir Dar, Amhara	Felege Hiwot Referral Hospital	Cross-sectional	NSNS	236	44.9 (38.6,51.2)
7	Negash A. et al. [47]	2014	Bahir Dar, Amhara	Felege Hiwot Referral Hospital	Cross-sectional	NSNS	373	67.1 (62.3,71.9)
8	Sharew N. et al. [48]	2018	Debre Birhan, Amhara	Debre Birhan Referral Hospital	Cross-sectional	NSNS	384	49.2 (44.2,54.2)
9	Alemu S. et al. [45]	2014	Debre Markos, Amhara	Debre Markos Referral Hospital	Cross-sectional	NSNS	392	56.9 (52.0,61.9)
10	Haile E. et al. [46]	2016	Dessie, Amhara	Dessie Referral Hospital	Cross-sectional	NSNS	374	52.5 (47.4,57.6)
11	Mensa M. et al. [28]	2017	Arba Minch, SNNP	Arba Minch General Hospital	Cross-sectional	NSNS	236	40.9 (35.5,46.3)
12	Legesse M. et al. [53]	2016	Hawassa SNNP	Hawassa University Specialized Hospital	Cross-sectional	NSNS	406	47.0 (42.2,51.9)
13	Jiru T. et al. [50]	2017	Nagele Borena, Oromia	Nagele Borena And Adola General Hospital	Cross-sectional	IPSQ	413	55.9 (51.1,60.7)
14	Ahmed T. et al. [18]	2014	Harar, Harari	Public Hospitals in Eastern Ethiopia	Cross-sectional	IPSQ	582	52.7 (48.6,56.8)
15	Molla T. [54]	2017	Mekelle, Tigray	Ayder Specialized Hospital	Cross-sectional	PPSNS	374	50.3 (45.2,55.4)

### Patient satisfaction with nursing care

**Fig. 2:**
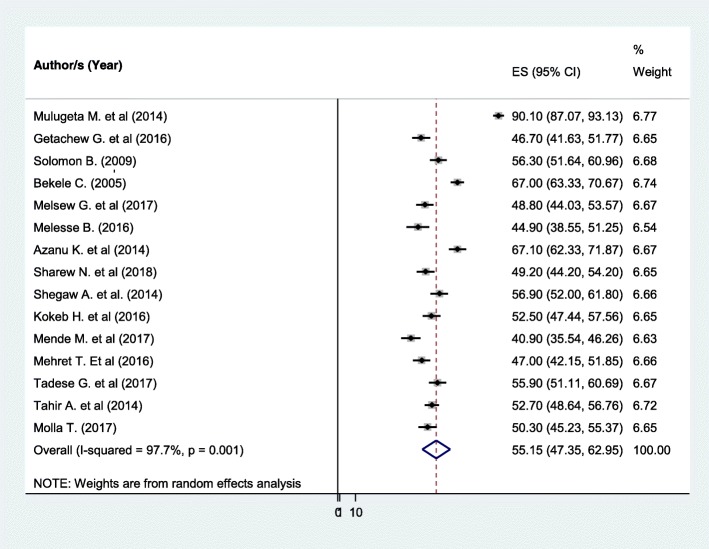
Forest plot showing the pooled level of satisfied patient with nursing care

The pooled effect size of patient satisfaction with nursing care using the fixed effect model showed significant heterogeneity across the studies. Therefore, we performed the analysis with a random effects model with 95% CI in order to adjust for the observed variability. Accordingly, the pooled national level of patient satisfaction with nursing care was 55.15% (95% CI (47.35, 62.95%)) with significant heterogeneity between studies (I^2^ = 97.7, *P* = 0.001) (Fig. 2).

**Fig. 3:**
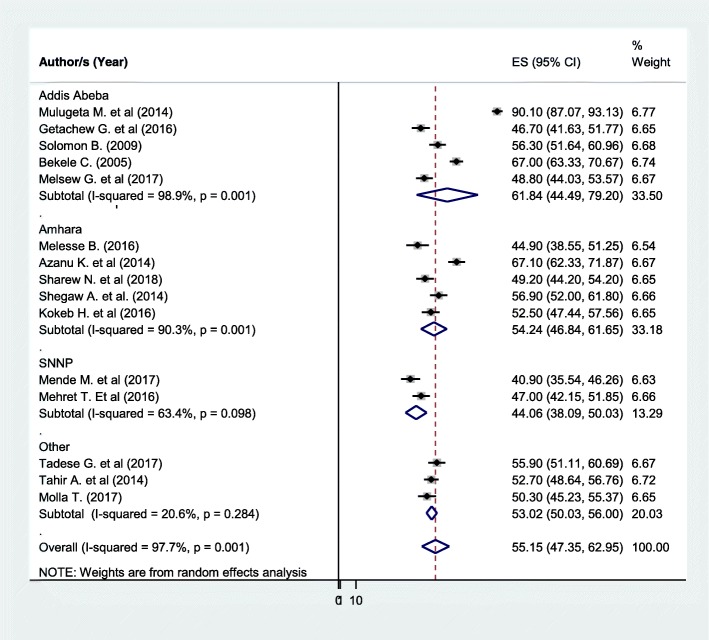
Subgroup analysis by regions on the level of patient satisfaction with nursing care

Based on the subgroup analysis by region, the highest level of patient satisfaction was observed in Addis Ababa (61.84% (95% CI: 44.49, 79.2), *I*^2^ = 98.9%) while, the lowest level of patient satisfaction was observed in SNNP region (44.06% (95% CI: 38.09, 50.03), I^2^ = 63.4%) (Fig. 3).

### Heterogeneity and publication bias

Given that the result of this meta-analysis revealed statistically significant heterogeneity among studies (I^2^ = 97.7%), we performed a subgroup analysis by region to adjust and minimize heterogeneity (Fig. 3). Furthermore, to identify the possible source of heterogeneity, we performed meta-regression analysis using sample size and publication year as covariates. However, none of them significantly affected heterogeneity between studies (Table 2).

**Table 2:** Meta-regression analysis of factors affecting between-study heterogeneity

Heterogeneity source	Coefficients	Std. Err.	*P*-value
Publication Year	−1.38	5.74	0.81
Sample size	0.02	0.19	0.94

**Fig. 4:**
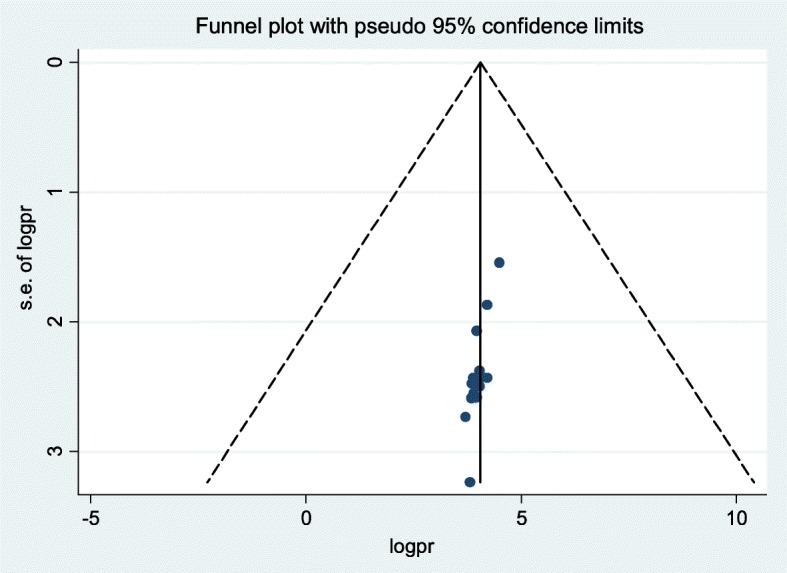
Funnel plot to test the publication bias in 15 studies

**Fig. 5:**
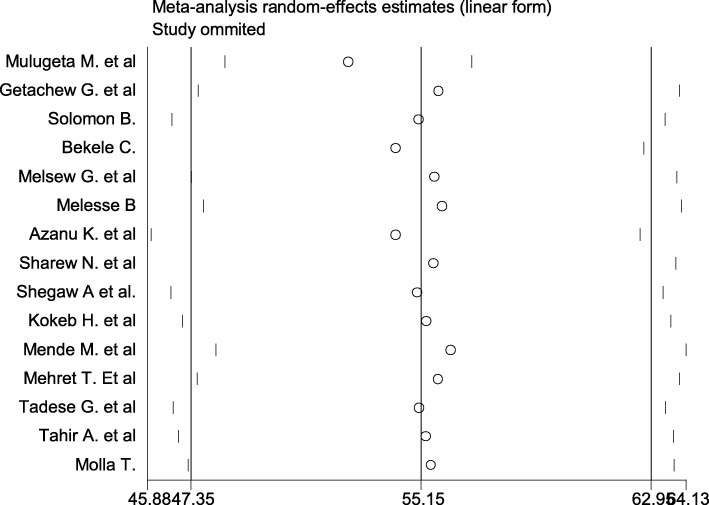
Result of sensitivity analysis of the 15 studies

Presence of publication bias was examined using visual inspection of the funnel plot and Egger’s test. Visual inspection of the funnel plot suggested symmetrical distribution of included studies (Fig. 4). However, the result of Egger’s test was statistically significant for the presence of publication bias (*P* = 0.001). Moreover, the result of sensitivity analyses using random-effects model suggested that none of the studies influenced the overall estimate (Fig. 5).

### Determinant factors associated with patient satisfaction

#### Availability of assigned nurse in charge of individual care

**Fig. 6:**
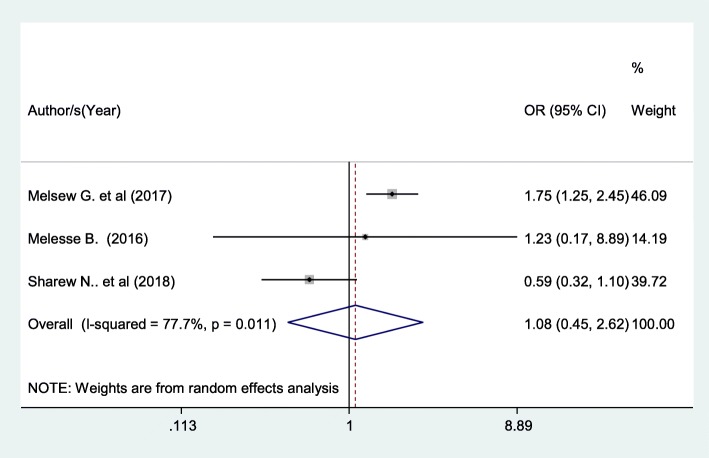
Forest plot showing the association between patient satisfaction and availability of an assigned nurse in charge of patient care

Patients who had one nurse in charge of their care had 8% higher chance of being satisfied with nursing care compared with those patients without the assigned nurse in charge of their care although not statistically significant (OR: 1.08 (95% CI (0.45,2.62), I^2^: 77.7%) (Fig. 6). The heterogeneity test (*P* = 0.011) showed significant evidence of variation across studies. The result of Egger’s test showed no statistically significant publication bias (*P* = 0.54).

### Place of residence

**Fig. 7:**
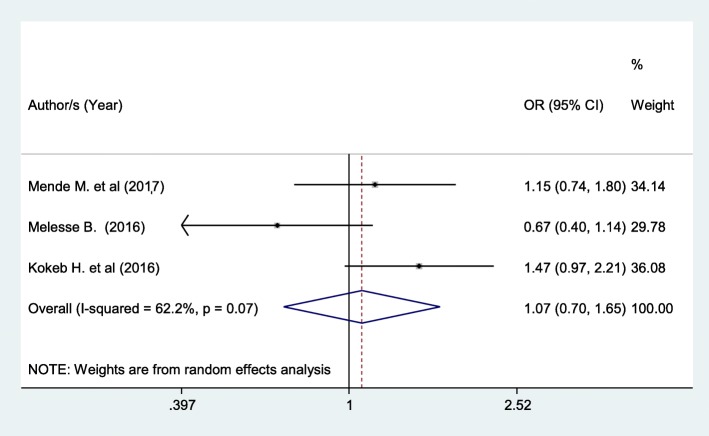
Forest plot showing the association between patient satisfaction and residence

Patients living in urban area had 7% higher chance of being satisfied with nursing care compared with those patients in a rural area although not statistically significant (OR: 1.07 (95% CI (0.70, 1.65), I^2^: 62.2%) (Fig. 7). The heterogeneity test (*P* = 0.07) showed no significant variation across studies. The result of Egger’s test showed significant evidence of publication bias (*P* = 0.01).

### History of admission

**Fig. 8:**
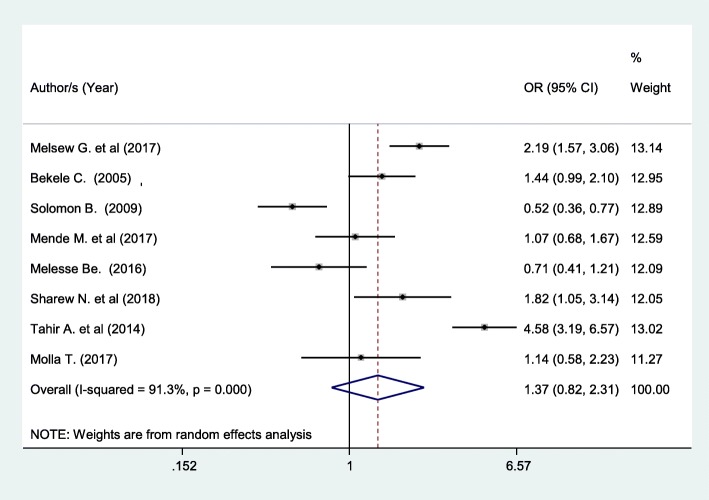
Forest plot showing the association between patient satisfaction and history of admission

Patients who had no history of previous hospitalization had 13.7% higher chance of being satisfied with nursing care compared with those patients with a history of hospitalization although not statistically significant (OR: 1.37 (95% CI (0.82,2.31), I^2^: 91.3%) (Fig. 8). The heterogeneity test (*P* = 0.001) showed a significant variation across studies. The result of Egger’s test showed no statistically significant evidence of publication bias (*P* = 0.25).

### Presence of comorbid diseases

**Fig. 9:**
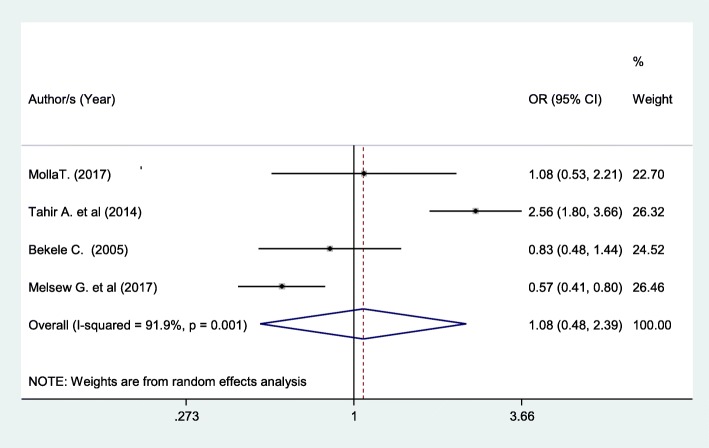
Forest plot showing the association between patient satisfaction and presence of comorbid diseases

Patients who had no comorbid disease had 8% higher chance of being satisfied with nursing care compared to those patients without comorbidity (OR: 1.08 (95% CI (0.48, 2.39), I^2^: 91.9%) although not statistically significant (Fig. 9). The heterogeneity test (*P* = 0.001) showed a significant variation across studies. The result of Egger’s test showed no statistically significant evidence of publication bias (*P* = 0.91).

## Discussion

Within the current scheme of healthcare, nurses spend their time more than any other healthcare professionals in the hospital by giving bedside nursing care for admitted patients. Patient satisfaction with nursing care is an indicator of good patient prognosis and a definitive determinant of the quality of healthcare in hospital [20, 58–60]. Hence, assessing the level of patient satisfaction with nursing care is crucial for improving the quality of care [61]. This meta-analysis was conducted to determine the national level of patient satisfaction with nursing care and identify factors associated with it using published and unpublished studies. The result of this meta-analysis revealed that the pooled national level of patient satisfaction with nursing care was 55.15% in Ethiopia. This finding was similar to previous studies conducted in Serbia (51.7%) and Philippines (57.8%) [62, 63]. However, the estimate of patient satisfaction with nursing care in our meta-analysis was lower than other similar studies report in Iran (69%) [23], Kenya (67%) [24], Jordan (69.4%) [55], Malaysia (82.7%) [3], and Saudi Arabia (89.6%) [16]. This could be due to poor job satisfaction, low standard of health care service and inadequate experience of nurses in Ethiopia. On the other hand, the level of patient satisfaction with nursing care in this meta-analysis was higher than study reports in Ghana (33%) [25] and Iraq (40 to 49%) [64]. The difference might be due to variation in sociodemographic characteristics of the study participants, sample size, and measurement tools used to quantify the level of satisfaction.

Our subgroup analysis by region revealed that the highest level of patient satisfaction was reported in Addis Ababa whereas the lowest was reported in SNNP region. This might be due to higher nurse to patient ratio and presence of experienced nurses and a high standard of nursing service in Addis Ababa as compared to the other regions of the country.

The result of this meta-analysis has found that patients’ residence, availability of an assigned nurse in charge, previous history of admission, and the presence of comorbid diseases had an influence on the patients’ satisfaction with nursing care even though not statistically significant. This result was in agreement with previous studies [64, 65]. Poor quality of care, repeated costs, and bad experience during their past admission might be the possible reasons for patients with a history of admission to be dissatisfied with nursing care. In parallel, a similar result was seen from another study where the availability of nurse in charge increases patients level of satisfaction with nursing care [66]. The possible reason might be due to the fact that availability of an assigned nurse in charge could help the patients to get a quick response for their needs and demand. Moreover, urban patients were more satisfied than rural patients. This might be due to well aware of the hospital service and their expectation was low as a result. The non-significant association in our meta-analysis might be due to the small number of studies used to estimate the pooled effect size.

Even though this review has provided valuable information and up-to-date evidence on the level of patient satisfaction with nursing care in Ethiopia, there were some limitations, which we address below. First, our overall estimates showed significant heterogeneity among studies, so that interpretation of the result has to be taken cautiously. Although we performed subgroup analysis and meta-regression, we could not identify the sources of heterogeneity. This might be due to high sensitiveness of Cochran’s Q test to the small number of studies included in the meta-analysis. Second, we only examined the influence of four factors because other major factors were not consistently investigated across the included studies. Third, it was difficult to compare our results with other national evidence due to the lack of published systematic reviews and meta-analysis on patient satisfaction with nursing. Finally, publication bias was detected in some of the estimates even though it is inevitable in any meta-analysis.

The findings of this meta-analysis have implication for clinical practice. It is known that patient satisfaction with nursing care is an indicator of the quality of care and nurses are the primary professionals to ensure optimal patient satisfaction and quality of nursing care. Therefore, determining the level of patient satisfaction with nursing care has implication to assist nurses to improve the quality of nursing care. This meta-analysis also indicated the influence of some factors on patient satisfaction that nurses should target during their routine nursing practice.

### Conclusions and recommendations

This systematic review and meta-analysis revealed that about one in two patients were not satisfied with the nursing care provided in Ethiopia. Even though the association was not statistically significant, patient satisfaction was influenced by patients’ history of admission, residence, availability of assigned nurse, and presence of comorbid diseases. This national evidence would be helpful for cross-country and cross-cultural comparisons in patient satisfaction level and factors influencing satisfaction in low- and middle-income countries. This might also be very useful for healthcare policymakers (e.g. Federal Ministry of health, Hospital administrators) to emphasize the quality of nursing care and to improve the overall quality of healthcare service at large. Given the multifactorial nature of factors influencing patient satisfaction with nursing care, further research is needed to identify additional factors especially from the patient’s perspective and explore context-specific strategies in order to increase the quality of nursing care.

## Data Availability

All the data are available from the corresponding author up on a reasonable request.

## Abbreviations

CI: Confidence Interval
IPSQ: Inpatient patient satisfaction questionnaire
NSNS: Newcastle satisfaction with nursing care scale
OR: Odds Ratio
PPSNS: Patient perception and satisfaction of nursing care scale
PRISMA: Preferred Reporting Items for Systematic Reviews and Meta-Analyses
SNNP: Southern Nations, Nationalities, and Peoples
